# Supplementary material for: Population-scale detection of methylation outliers from long-read genome sequencing
Source: medRxiv. 2026 Jun 11:2026.06.09.26355279. Preprint. [Version 1] doi: 10.64898/2026.06.09.26355279 (PMC13278280; doi:10.64898/2026.06.09.26355279)
Supplement: Supplementary file 1 [file NIHPP2026.06.09.26355279V1-supplement-1.pdf]

medRxiv preprint doi: https://doi.org/10.64898/2026.06.09.26355279; this version posted June 11, 2026. The copyright holder for this preprint
(which was not certified by peer review) is the author/funder, who has granted medRxiv a license to display the preprint in perpetuity.
It is made available under a CC-BY 4.0 International license .

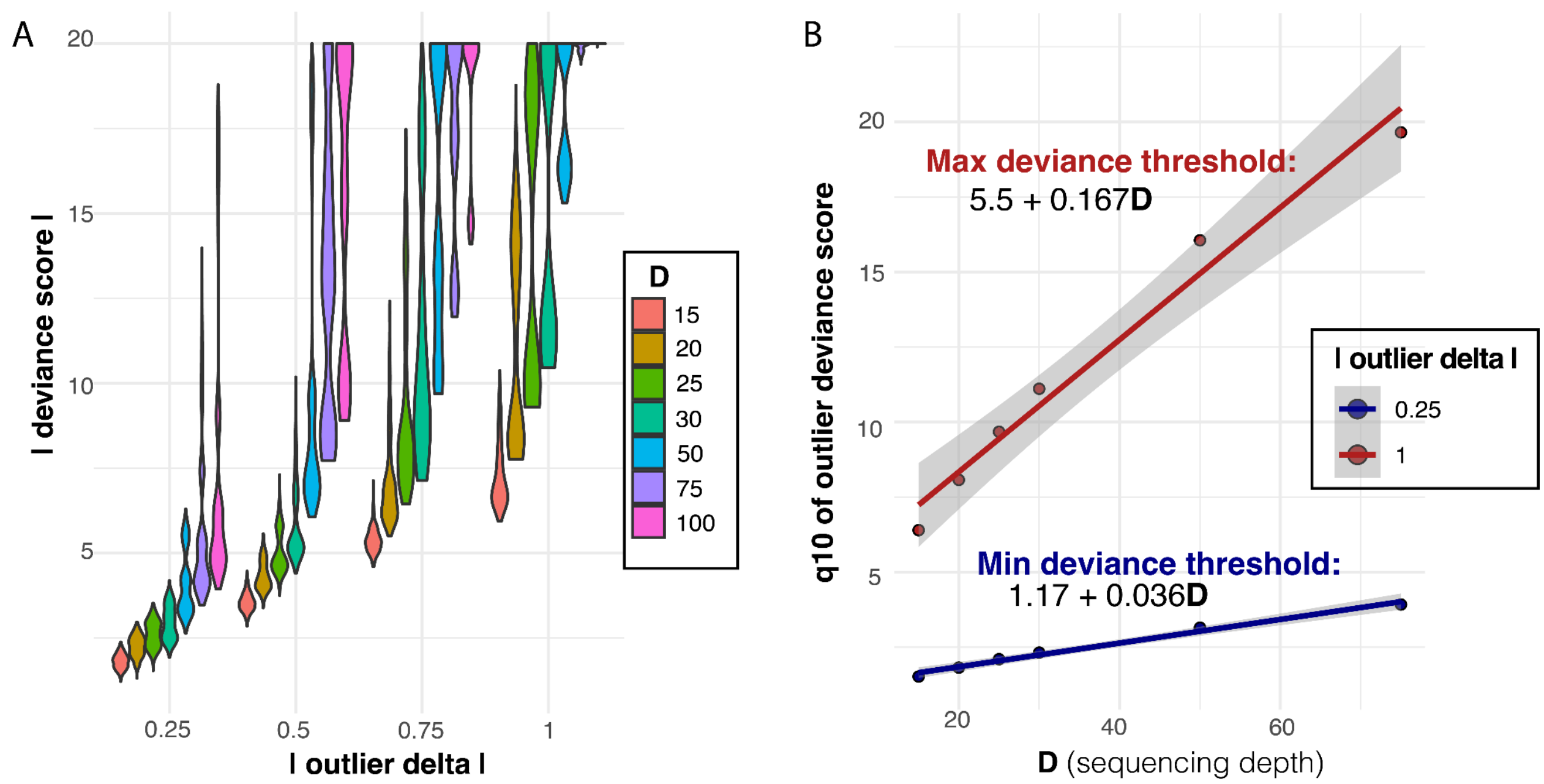

Supplemental Figure 1: learning thresholds for deviance score from simulated outliers. **A** Distribution of deviance scores at various outlier deltas for simulated outliers across a range of depth thresholds. As depth increases so do absolute deviance scores. **B** Linear relationship between depth and the 10th quantile of observed deviance score from simulated outliers. Q10 at an absolute delta of 0.25 is set as a minimum deviance score threshold to ensure 90% power at detecting outliers of magnitude 0.25. Q10 at an absolute delta of 1.0 is set as maximum deviance score to avoid extreme inflation and infinite values of deviance score. Slope and intercept for these depth-dependent thresholds are annotated from fitting linear models.

medRxiv preprint doi: https://doi.org/10.64898/2026.06.09.26355279; this version posted June 11, 2026. The copyright holder for this preprint (which was not certified by peer review) is the author/funder, who has granted medRxiv a license to display the preprint in perpetuity.
It is made available under a CC-BY 4.0 International license .

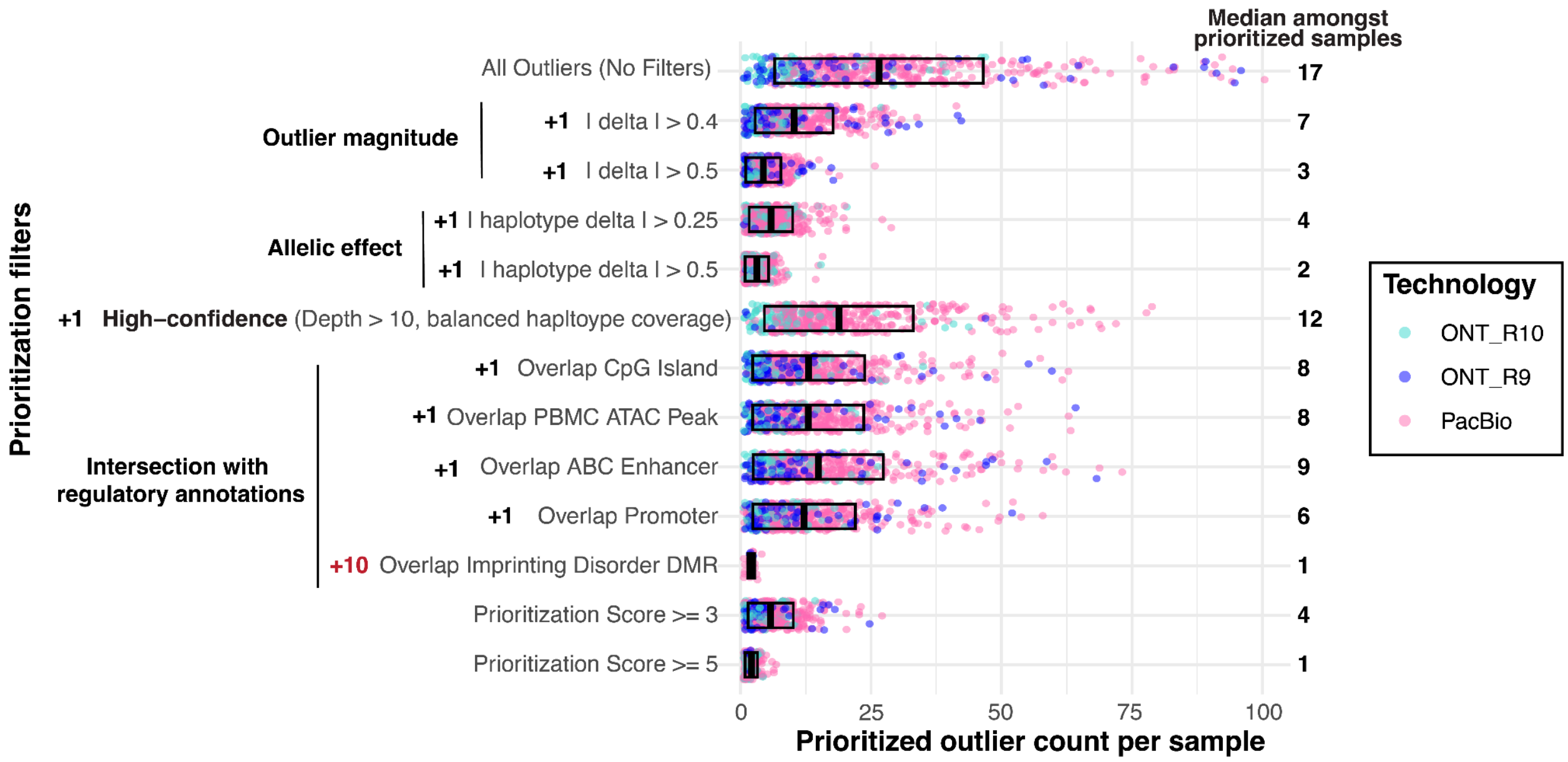

Supplemental Figure 2 Outlier prioritization scores. Outlier prioritization from METAFORA applied to GREGoR U08 and UDN long-read data. X-axis displays the number of outliers per sample that meet the corresponding prioritization filter on Y-axis, colored by sequencing technology. Outliers gain the specified number of points for meeting filter criteria. Median outlier count per sample annotated for each filter, amongst samples with at least one outlier meeting filter. At the end of scoring, samples had a median of 4 outliers with a prioritization score of at least 3 and 1 outlier with prioritization score of at least 5.

medRxiv preprint doi: https://doi.org/10.64898/2026.06.09.26355279; this version posted June 11, 2026. The copyright holder for this preprint (which was not certified by peer review) is the author/funder, who has granted medRxiv a license to display the preprint in perpetuity.
It is made available under a CC-BY 4.0 International license .

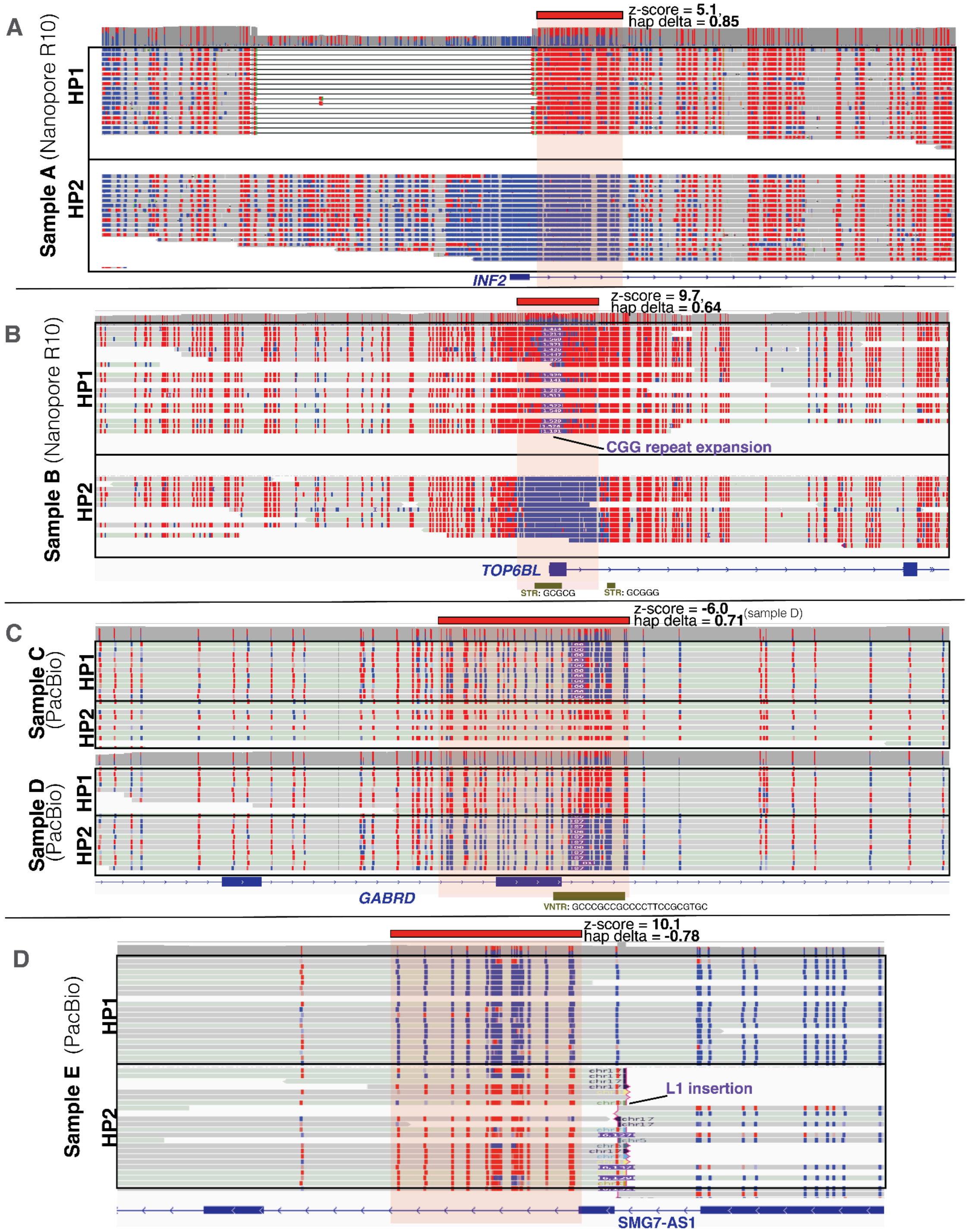

Supplementary Figure 3. **IGV screenshots displaying high haplotype delta outliers near rare structural variants. A** 1.7Kb deletion of the promoter region of *INF2* observed upstream of a hypermethylation outlier in a nanopore R10 sample. **B** 1.5Kb CGG repeat expansion insertion in promoter/5'UTR of *TOP6BL* gene phased together with a hypermethylation outlier in a Nanopore R10 sample. **C** Rare insertions in a VNTR region intronic to the *GABRD* identified in multiple samples. Sample C sequenced from PacBio has 3 additional copies of 22bp repeat motif (z-score=-4.52), and is associated with hypomethylation of that haplotype. Sample D also from PacBio has additional 4 copies and even stronger hypomethylation (z-score=-5.99). **D** A rare 6Kb L1 retrotransposon insertion identified and phased together with an upstream hypermethylation outlier of an *SMG7-AS1* intron in a PacBio sample. In all IGV screenshots gene models are displayed below along with adotto catalog of tandem repeat loci and their annotated motif.

medRxiv preprint doi: https://doi.org/10.64898/2026.06.09.26355279; this version posted June 11, 2026. The copyright holder for this preprint (which was not certified by peer review) is the author/funder, who has granted medRxiv a license to display the preprint in perpetuity.
It is made available under a CC-BY 4.0 International license .

Supplementary Methods

Outlier simulation

Outliers were simulated across a large range of parameters following the code in "outlier_simulation_benchmark/outlier_simulation.R". In brief, we used mean population betas

medRxiv preprint doi: https://doi.org/10.64898/2026.06.09.26355279; this version posted June 11, 2026. The copyright holder for this preprint (which was not certified by peer review) is the author/funder, who has granted medRxiv a license to display the preprint in perpetuity.
It is made available under a CC-BY 4.0 International license .

across chr1 as a background. We selected a random CpG within the interior of the chromosome and flanked that site on either side by a specified number of CpGs given by the ***context_length*** parameter (default: 1,500). Into this region, we spiked in an outlier segment of length ***M*** (default: 20 CpGs), and set the expected methylation beta at these CpGs to the specified ***pop_median*** parameter, with additional variance introduced by the **noise** parameter to reflect small deviations among consecutive CpGs. This profile defines the expected "true" population methylation levels for control samples.

We then simulate a single outlier sample, by adding an outlier ***delta*** parameter to all CpGs within the designated outlier segment to give us the expected outlier methylation proportions for the region. Methylation betas for controls and the outlier sample were winsorized to the range [*1/D, (1-D)/D*], where ***D*** is the depth parameter, to prevent beta values reaching extremes of greater than one or less than zero. These vectors define the expected "true" population betas for both control samples and the outlier sample. To simulate observed betas, methylation proportion values were drawn from a beta distribution with shape parameters determined by the expected population beta and the simulated CpG depth for each of the *N-1* controls and the 1 outlier sample. To simulate CpG depths, values were drawn from a Poisson distribution parameterized by the overall mean depth parameter ***D*** across all CpGs and samples. To account for technical sources of noise in estimating observed betas, we additionally added Gaussian noise scaled by the ***noise*** parameter to the observed betas.

We simulated beta and depth matrices for all control samples and the outlier sample across a range of parameters for population median beta, outlier delta, ***N*** (number of samples), ***M*** (number of CpGs in the outlier segment), ***D*** (mean sequencing depth), and the noise parameter. We then ran METAFORA outlier calling on 10 replicates for each combination of parameters. To evaluate outlier detection performance, we calculated the overlap between outlier regions identified by METAFORA and the expected true outlier coordinates. Outlier regions with no overlap with the true outlier were classified as false positives. Outlier regions with at least 50% reciprocal overlap with the true outlier were classified as true positives.

Methylation technology comparison

To compare genome-wide estimation of methylation proportions across different long-read sequencing platforms, we collated long-read data from the Undiagnosed Disease Network and GREGoR consortium. BAM files containing methylation marks were downloaded from UDN and

medRxiv preprint doi: https://doi.org/10.64898/2026.06.09.26355279; this version posted June 11, 2026. The copyright holder for this preprint (which was not certified by peer review) is the author/funder, who has granted medRxiv a license to display the preprint in perpetuity.
It is made available under a CC-BY 4.0 International license .

GREGoR dbGaP under the following accession numbers (phs003047,phs001232). For the technology comparison section we restricted to samples sequenced from whole blood, which included 77 ONT R9 samples (68 sequenced from Stanford UDN, 9 sequenced from UW GREGoR site), 98 ONT R10 samples (8 sequenced from Stanford UDN, 15 sequenced from UW GREGoR site, 17 sequenced from UCI GREGoR site, and 58 sequenced from Broad GREGoR site), and 376 PacBio samples (286 sequenced from Stanford GREGoR site, 90 sequenced from UCI GREGoR site).

METAFORA processing (see METAFORA methods 1) were applied on each bam file according to whether it was PacBio or ONT to get genome-wide methylation beta and depth matrices for each sample. Segmentation algorithm was performed as described in METAFORA Methods 2.2, to segment genome-wide CpG methylation profiles into correlated blocks of contiguous CpGs to reduce dimensionality. These CpG blocks were used to identify and exclude 10 global outlier samples based on low mean pairwise correlations < 0.95, including 3 ONT R9 samples with low coverage (<15x) and 7 ONT R10 samples from Broad's GREGoR site that were potentially misannotated and sequenced from a tissue other than whole blood (such as PBMCs or saliva). For each technology the mean beta across all technology samples was calculated as well as the mean effective depth, number of reads where methylation was confidently called. Segments were labeled based on mean segment beta across all samples as either hypomethylated (beta < 0.25), hypermethylated (beta > 0.75), or otherwise variably methylated. PCA was also performed on the segment beta matrix using the pcatools package in R.

For the mitochondrial error rate estimation, we first filtered long-read bams to only primary alignments aligned to chrM, removing supplementary and non-primary reads that could reflect nuclear mitochondrial DNA insertions. From these filtered bams, we calculated methylation beta and depth matrices for all samples, and then assigned the estimated error rate to be the percentage of reference methylated CpGs over all primary alignments, the main assumption being mitochondrial DNA should not have any CpG methylation. These estimated error rates were then summarized and compared across sequencing technologies.

To find differentially methylated segments between PacBio and ONT, we first excluded R9 samples to filter down to 376 PacBio samples and 91 R10 samples. We used the R package limma (lmFit) to run a linear model for each autosomal methylation segment to predict M-transformed methylation levels on an indicator variable for if the sample was sequenced on

medRxiv preprint doi: https://doi.org/10.64898/2026.06.09.26355279; this version posted June 11, 2026. The copyright holder for this preprint (which was not certified by peer review) is the author/funder, who has granted medRxiv a license to display the preprint in perpetuity.
It is made available under a CC-BY 4.0 International license .

PacBio. We then labeled each segment based on the absolute difference between the median ONT beta and the median PacBio beta. Significantly deviating segments were defined as those that had an absolute technology difference of greater than 0.1 (10 percentage points difference in either technology).

Outlier characterization and enrichment analysis

To test METAFORA on real world data, we used long-read data from UDN and GREGoR as described above as input into METAFORA. We ran METAFORA with minimum outlier size of 30 CpGs and minimum absolute outlier delta of 0.3 to focus on the largest effect deviations. We calculated burden of outliers per genome and removed 10 samples who were global outliers (>165 outliers per genome, corresponding to a scaled outlier burden z-score of 3). To functionally characterize outlier regions that were output by METAFORA, we first created a background set of non-outlier regions by sampling 100,000 segments from the genome wide population mean segmentation. These background regions were then compared to outlier segments based on their length and mean beta across all samples. To assess noncoding constraint, gnocchi non-coding constraints z-scores for 1kb bins of the genome were downloaded from
https://hgdownload.soe.ucsc.edu/gbdb/hg38/gnomAD/mutConstraint/mutConstraint.bw (46). Segments were summarized as the mean gnocchi z over all overlapping bins.

To compute segment-level enrichments based on functional annotations, we compiled a list of functional annotations. These included CpGIslandsExt from UCSC table browser https://genome-euro.ucsc.edu/cgi-bin/hgTables, for CpG Islands. These islands were flanked on either side by 2Kb to define CpG shores. And additionally flanked an additional 2Kb from these shores to define CpG shelfs. ABC enhancers from all cell types were downloaded from following FTP server
(https://mitra.stanford.edu/engreitz/oak/public/Nasser2021/AllPredictions.AvgHiC.ABC0.015.minus150.ForABCPaperV3.txt.gz). LiftOver was used to map these hg19 coordinates onto hg38, and then enhancers with the same target gene and type (promoter, genic, intergenic) were merged across also cell type. The Merged GRCh38 coordinate ABC enhancers can be downloaded here
(https://github.com/tjense25/METAFORA/blob/main/references/ABC_enhancers.merged.GRCh38.bed.gz) (40,69). GREGoR PBMC ATAC data was joint called using ENCODE pipeline to define PBMC open chromatin, available for download here

medRxiv preprint doi: https://doi.org/10.64898/2026.06.09.26355279; this version posted June 11, 2026. The copyright holder for this preprint (which was not certified by peer review) is the author/funder, who has granted medRxiv a license to display the preprint in perpetuity.
It is made available under a CC-BY 4.0 International license .

(https://github.com/tjense25/METAFORA/raw/main/references/GSS_PBMC_ATACpeaks.open_chromatin.GRCh38.bed.gz) (70). Finally, ENCODE cis conserved regulatory elements (cCRE) were downloaded from UCSC table browser https://genome.ucsc.edu/cgi-bin/hgTrackUi?db=mm10&g=encodeCcreCombined (ENCODE).

We label our outlier and background segments based on if it overlaps each annotation (plyranges overlapsAny function). To calculate enrichments, we then apply fisher's exact test to get odds ratios, confidence intervals, and p-values.

Multi-omics enrichments

To calculate enrichments across multiple omics layers, we use additional multi-omics data available from UDN and GREGoR as described below. We computed these enrichments at the gene level to make it consistent across the different layers of regulation. To define methylation outlier genes, we took gene coordinates from GENCODE v32, subset to protein coding genes, slopped them with an additional 10Kb on either side, and then intersected with methylation outlier coordinates from METAFORA output for the 494 blood tissue samples. The gene in each sample was labeled binary as containing an outlier nearby or not. To compute pairwise multi-omics enrichments we then subset to samples present in both assays, and subset to genes that had at least one outlier sample in both assays. Fisher's exact test was then used on gene counts across the contingency table of outlier presence across each of the two omes to get enrichment odds ratio, confidence interval, and p-value.

Rare SVs

We use the same 551 UDN and GREGoR input genomes to METAFORA as input to call structural variants. Sniffles2 (71) was called on each long-read sample bam using recommended default parameters. Resulting SV vcf files were merged with jasmine as previously described (42,72). From the joint SV file, allele frequencies were estimated from observed allele counts and then variants filtered to those with minor allele frequency < 1%. Breakends (SVTYPE = "BND") and SV calls smaller than 50bp were also filtered out. As quality control, we removed 6 samples which contained an excess number of breakends or SVs (defined as samples with > 5 sd above mean count per genome for any SV type). We intersected rare SV coordinates with slopped gene coordinates to define rare SV genes. In the SV-methylome enrichment, we calculated enrichment based on 484 shared samples across 4,575 shared genes.

medRxiv preprint doi: https://doi.org/10.64898/2026.06.09.26355279; this version posted June 11, 2026. The copyright holder for this preprint (which was not certified by peer review) is the author/funder, who has granted medRxiv a license to display the preprint in perpetuity.
It is made available under a CC-BY 4.0 International license .

ATAC-seq Chromatin Accessibility Outliers

To call chromatin accessibility outliers, we followed methods recommended by EpiOut (8). First, we started with ATAC bam files for 184 GREGoR participants processed with the ENCODE pipeline (70). We downsampled and then merged reads from all bams into a single monolithic bam file. This file was then run through MACS2 peak calling to define a joint peak set. This joint peak set was then intersected with sample-level bams to get a count matrix across all samples and peaks. This count matrix was then used as input to EpiOut with default parameters to yield chromatin accessibility outliers. EpiOut reports log2 fold change of each ATAC peak defined as log2 ratio of observed counts divided by an autoencoder-corrected predicted expected count. EpiOut also reports a p-value per peak that this fold change deviance is significantly different from the null hypothesis. We define outliers as peaks where the multiple-testing corrected p-value, *padj*, is less than alpha threshold 0.05. As quality control, we removed 2 samples that displayed a higher than expected number of outliers per genome (defined as > 3sd above mean expected burden of outliers). We intersected outlier peak coordinates with slopped gene coordinates to define ATAC outlier genes. In the ATAC-methylome enrichment, we calculated enrichment based on 155 shared samples across 1,058 shared protein-coding genes.

RNA-seq Expression Outliers

To call expression outliers we followed the same procedure as previously described for RNA-seq data (42,73). We started with 455 GREGoR and UDN RNA bam files sequenced from whole blood aligned to hg38 with STAR. Gene counts were quantified with RNASeQC, corrected for library size and composition, and then log-normalized. PCA was used to estimate hidden factors, and log-scaled counts were then residualized on these top 50 PCs, along with sex and sequencing batch indicator variables. Finally, residuals per gene were centered and scaled to get expression z-scores. Expression outliers were defined as any gene with absolute z-score > 2. As quality control, we removed 9 samples that were global outliers, containing an excessive burden of outliers (>3sd above mean expected burden of outliers per genome). In the RNA-methylome enrichment, we calculated enrichment based on 108 shared samples across 3,832 shared protein-coding genes.

For isoform-level expression z-scores in the GNAS example, a similar process was performed but using RSEM to quantify genes based on STAR transcriptome-aligned bams. Specific GNAS transcript *ENST00000485673* was chosen for analysis as its TSS overlaps imprinting and outlier region and it contained unique exonic sequence to enable isoform quantification.

medRxiv preprint doi: https://doi.org/10.64898/2026.06.09.26355279; this version posted June 11, 2026. The copyright holder for this preprint (which was not certified by peer review) is the author/funder, who has granted medRxiv a license to display the preprint in perpetuity.
It is made available under a CC-BY 4.0 International license .

OLink Proteomics Outliers

To call proteomics outliers, we used plasma protein abundance data from Olink HT assay available for 172 GREGoR participants. To normalize data per protein, we started with PCNormalizedNPX values generated through Olink's workflow. These NPX values are normalized intensities to plate controls that are log-scaled and residualized on PCs to correct for additional technical factors. GREGoR sample proteomics were generated from 3 separate plates, so to further correct potential batch effects, we performed additional PC hidden factor residualization. PCA was performed on the matrix of PCNormalizedNPX values across all samples, and top 20 PCs along with indicator values for the different plateID were corrected with a linear model. Residuals were then scaled and centered to obtain protein z-scores. We defined protein outliers as a protein with absolute z-score > 2. To assign proteins to genes, UniProt IDs provided by assay were mapped to ENSEMBL gene ids and hgnc symbols using biomart. We did not observe any major global outliers in the proteomics data in terms of outlier burden per genome. In the Protein-methylome enrichment, we calculated enrichment based on 120 shared samples across 1,361 shared protein-coding genes.
